# The One-Year In Vivo Comparison of Lithium Disilicate and Zirconium Dioxide Inlays

**DOI:** 10.3390/ma14113102

**Published:** 2021-06-05

**Authors:** Rini Behera, Lora Mishra, Darshan Devang Divakar, Abdulaziz A. Al-Kheraif, Naomi Ranjan Singh, Monika Lukomska-Szymanska

**Affiliations:** 1 Department of Conservative Dentistry & Endodontics, Institute of Dental Sciences, Siksha ’O’ Anusandhan, Bhubaneswar P.O. Box 751003, India; rinibehera@soa.ac.in (R.B.); loramishra@soa.ac.in (L.M.); naomiranjansingh@soa.ac.in (N.R.S.); 2 Dental Biomaterials Research Chair, Department of Health Department, College of Applied Medical Sciences, King Saud University, Riyadh P.O. Box 10219, Saudi Arabia; ddivakar@ksu.edu.sa (D.D.D.); aalkhuraif@ksu.edu.sa (A.A.A.-K.); 3 Department of General Dentistry, Medical University of Lodz, 251 Pomorska St, 92-213 Lodz, Poland

**Keywords:** CAD-CAM, inlay, lithium disilicate, zirconium dioxide

## Abstract

The objective of the present study was to evaluate the one-year clinical performance of lithium disilicate (LD) and zirconium dioxide (ZrO_2_) class II inlay restorations. Thirty healthy individuals who met the inclusion criteria were enrolled for the study. The patients were randomly divided into two study groups (*n* = 15): LD (IPS e.max press) and ZrO_2_ (Dentcare Zirconia). In the ZrO_2_ group, the internal surfaces of the inlays were sandblasted and silanized with Monobond N (Ivoclar, Leichsteistein, Germany). In the LD group, the internal surfaces of the inlays were etched with 5% hydrofluoric acid. The ceramic inlays were cemented with self-cure resin cement (Multilink N). Clinical examinations were performed using modified United State Public Health Codes and Criteria (USPHS) after 2 weeks, 4 weeks, 6 months and 1 year. The one-year survival rate was evaluated. In total, one failure was observed in the ZrO_2_ group. The survival probability after 1 year for the ZrO_2_ inlays was 93%, and for the LD inlays was 100%, which was statistically insignificant. The differences between both groups for most USPHS criteria (except for colour match) were statistically insignificant. Within the imitations of the present study, the lithium disilicate- and zirconia dioxide-based inlays exhibited comparable clinical performances. However, the colour and translucency match was superior for the lithium disilicate restorations.

## 1. Introduction

The prevalence of dental caries is estimated by the WHO to be over 90% [1]. The extension of caries is the prime dominance factor in choice of reconstruction method. Currently, composite restorations, crowns, inlays or onlays are recommended to reconstruct extensive class II MOD cavities [2]. However, in these cases, the establishment of occlusal anatomy, proximal contact and the contour, finishing and polishing of indirect restorations are far superior to direct reconstructions [3].

Ceramic and zirconium dioxide-based reconstructions provide enhanced strength and aesthetics [3,4]. Both materials offer the opportunity to maintain the tooth structure while providing the mechanical benefits of modern adhesive technology. Lithium disilicate (LD) glass ceramic is excellent for highly aesthetic restorations providing good mechanical properties. LD ceramic, the strongest and the toughest of the glass-ceramics available, exhibits moderate flexural strength (360–440 MPa) [5] and fracture toughness (2.5–3 MPa m^1/2^) [6], yet provides excellent translucency and shade matching properties [7,8].

On the other hand, zirconium oxide (ZrO_2_) is largely used due to its favourable mechanical properties and good fracture resistance. The biocompatibility, optical properties and translucency of ZrO_2_ make it an alternative to porcelain-fused-to-metal restorations [3]. Additionally, ZrO_2_ is the strongest and most robust of all dental ceramics with a flexural strength of 800–1200 MPa and fracture toughness of 6–8 MPa m^1/2^ [4]. Therefore, it meets the mechanical requirements for high-stress bearing posterior restoration. Unfortunately, the limited translucency and poor adhesion to tooth structure, due to its inert and non-polar nature, are major disadvantages [9,10].

The survival rate of ceramic restorations has been largely investigated [8,11,12,13,14,15,16]. However, due to a lack of clinical studies, there is a great need to evaluate LD and ZrO_2_ inlays in in vivo studies. Therefore, the objective of the present study was to evaluate and compare the one-year clinical performance of LD and ZrO_2_ inlay restorations. The null hypothesis was that there is no difference in the survival rate and quality between LD inlay and ZrO_2_ inlay restorations.

## 2. Materials and Methods

### 2.1. Study Design

This research protocol and design was approved by the Institutional ethical committee (Ref. No/DMR/IMS.SH/SOA/180035). Thirty healthy individuals who met the inclusion criteria were enrolled for the study (Table 1) [16]. The patients were randomly divided into two groups (*n* = 15) with online software www.randomizer.org (first accessed on 28 May 2018) (Urbaniak, G. C., & Plous, S. 2013, Research Randomizer, Version 4.0, Computer software). The cavity distribution within the study groups is presented in Table 2. The distribution of the tooth and cavity type was not significant at *p* < 0.05.

### 2.2. Tooth Preparation

**Figure 1:**
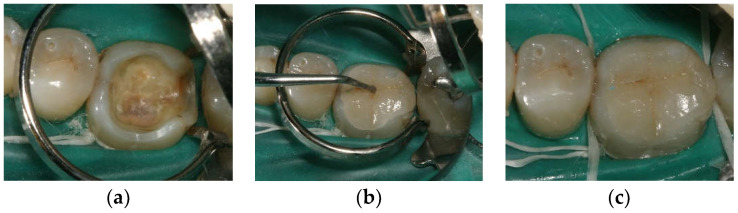
(**a**) The inlay preparation; lower molar, class IIOD cavity; (**b**) Try-in of the inlay; (**c**) Lithium disilicate inlay in situ.

After administering the local anaesthetic (Indoco, Warren Lignox with Adrenaline, Mumbai, India), class II cavities were prepared using a high-speed handpiece with inlay diamond points (Coltene Diatech Inlay & Crown preparation kit 11312, Altstätten, Switzerland) under a constant, copious water supply. The isthmus width was established at a minimum of 2.5 mm, the pulpal floor depth amounted up to at least 1.5–2.0 mm, the axial wall depth was up to 1.5 mm, the internal line angles were rounded and the divergence angle of the cavity was approximately 10°–15° with no bevel (Figure 1a). The enamel margins were refined using an enamel hatchet hand instrument (Hu-Friedy Mfg. Chicago, IL, USA).

### 2.3. Impressions

Gingival retraction was achieved with gel (Racegel, Septodont Saint Maur des Fosses, France) applied for 2 min.

#### 2.3.1. Zirconium Oxide (ZrO_2_) Group

In the zirconium oxide (ZrO_2_) group, teeth were digitally scanned with an intraoral scanner (CEREC Omnicam scanner; Dentsply Sirona, Bensheim, Germany), followed by conversion to a 3-dimensional (3D) virtual model (CEREC AC software 4.3; Dentsply Sirona, Charlotte, NC, USA). An irreversible hydrocolloid impression (Alginate, Zelgan Plus, Dentsply, Gurgaon, India) for the antagonist arch was taken and disinfected with 0.5% sodium hypochlorite solution. Antagonist impression casts were immediately poured with dental stone type IV (Durone, Dentsply, Petropolis, RJ, Brazil). The impressions scans and antagonist cast were sent to the laboratory for fabrication of the inlays.

#### 2.3.2. Lithium Disilicate (LD) Group

In the lithium disilicate (LD) glass ceramic group, full arch impressions (the two-step putty wash technique) with elastomeric putty impression material (Silagum-Putty, DMG, Germany) and light body impression material (Silagum-Light, DMG, Hamburg, Germany) using stock trays (GDC Dentulous Perforated Impression Trays, Hoshiarpur, Punjab, India) were taken. The impressions were disinfected for 10 min in glutaraldehyde (2%) solution and rinsed with water for 15 s. The antagonist arch impressions were taken and disinfected as described for the ZrO_2_ group. The impression and antagonist cast were sent to the laboratory for fabrication of the inlays.

### 2.4. Shade Selection, Occlusion Registration and Temporalization

For both groups, shades were selected from the Classical Vita shade guide (VITA Zahnfabrik, Germany) [17]. Occlusion registration was performed using bite registration wax (Denar^®^ Bite Registration Wax, Whip Mix Corp, Louisville, KY, USA). Patients were temporized with Orafil LC (Prevest, Brahmana, Jammu, India) until the delivery of the final inlay for one week.

### 2.5. Fabrication of Inlay

#### 2.5.1. ZrO_2_ Group

The master cast models were poured using type IV dental stone (Elite stone, Zhermack, Badia Polesine (RO), Italy). The inlay design and finish line marking were planned with CEREC AC 4.3 (Dentsply Sirona, Charlotte, NC, USA) software. The marginal discrepancy was set at 0.0 mm, and the margin thickness was at 0.2 mm. The simulated die spacer was programmed at 30 μm, starting 1.0 mm away from the margin [18]. This was followed by an assessment of the master cast model physically and virtually. The inlays were fabricated from monolithic zirconia (DentCare Zirconia, Weiland Zenostar, Ivoclar Vivadent, Pforzheim, Germany) in CORiTec 250i milling unit (imes-icore dental solutions, Eiterfeld, Germany).

The milled discs were manually separated from the zirconia blanks and sintered using Austromat μSiC furnace (Dekema, Freilassing, Germany) for 9 h at 1450 °C. Then, the inlays were glazed by applying Ivoclar glazing paste e-max (Ivoclar Vivadent, Liechtenstein, Germany), with the thickness ranging between 20 and 50 μm, and fired in a furnace (Ivoclar P310 furnace, Liechtenstein, Germany). The restorations were then mirror-finished with diamond-impregnated silicone instruments (Brasseler, Savannah, GA, USA) and polishing pastes (Perfect Polish, Henry Schein, Melville, NY, USA). Finally, the occlusion and proximal contacts were checked and adjusted on the master cast model using stereomicroscope 5× magnification (Labomed CZM6, Labo America Inc., Houston, TX, USA).

#### 2.5.2. LD Group

Master casts were poured using type IV stone gypsum (Elite stone, Zhermack, Badia Polesine (RO) Italy). The inlay wax patterns were fabricated and invested in a phosphate bonded investment, IPS PressVEST Speed (Ivoclar Vivadent, Schaan, Leichsteistein, Germany). The restorations were fabricated from lithium disilicate ingots (IPS e.max Press, Ivoclar Vivadent, Schaan, Leichsteistein, Germany) in a press furnace EP600 (Ivoclar Vivadent, Schaan, Leichsteistein, Germany) at 920 °C at 600 kPa pressure following the manufacturer’s recommendations with the lost-wax technique (spacer of 60 mm).

Glazing (IPS e.max Ceram Glaze Liquid, Ivoclar Vivadent, Schaan, Leichsteistein, Germany) firing was performed in a P200 furnace (Ivoclar Vivadent, Schaan, Leichsteistein, Germany). The restorations were adjusted with water cooled diamond rotary instruments (Set 4562, Brasseler GmbH, Savannah, GA, Germany). The internal surface of the restorations was sandblasted with 50-mm aluminium oxide particles at a pressure of 6 Bar (Opiblast, Buffalo Dental Mfg., Inc. Syosset, NY, USA). An initial assessment of the inlays on the master model with stereomicroscope (Labomed CZM6, Labo America Inc., Fremont, CA, USA) at 5× magnification was performed.

### 2.6. Clinical Try-In and Luting Procedure

The temporary restorations were removed using a probe. The inlays were carefully tried in under the split rubber dam isolation technique (Figure 1b). With the aid of OptraStick (Ivoclar, Vivadent, Lienchtenstein, Germany), the inlays were handled and securely positioned within the cavity, and the fit was evaluated. Next, the interproximal contacts and colour were examined. The tooth was cleaned with a slurry of ultrafine pumice and water and then air dried before luting.

In the ZrO_2_ group, the internal surfaces of the inlays were sandblasted with aluminium oxide particles. The surface was then silanized with Monobond N (Ivoclar, Leichsteistein, Germany). While, in the LD group, the internal surfaces of the inlays were etched with 5% hydrofluoric acid (IPS Ceramic Kit, Ivoclar, Leichsteistein, Germany) for 20 s, cleaned with water and dried. Self-etch adhesive luting cement (Multilink N-system, Ivoclar, Leichsteistein, Germany) was used according to the manufacturer’s recommendations. The restoration was then seated with slight pressure.

The excess resin cement was light cured (Mectron, Starlight P, Mectron Pvt Ltd., Karnataka, India) for 1–2 s for smooth excess removal (Figure 1c). Subsequently, additional light-curing for 20 s per surface was performed. Margins of luted restorations were refined using fine round tapered diamond burs (MANI Diamond Burs, CR series, Takanezawa factory, Shioya, Tochigi, Japan) and rubber points (Brasseler, Savannah, GA, USA) under water cooling. After removal of the rubber dam, the occlusal contacts were checked, and interferences were removed. Next, final finishing and polishing was performed.

The bitewing and intraoral periapical radiograph (IOPA) of the cemented inlays in both groups were taken to assess the immediate post-op marginal adaptation.

### 2.7. Evaluation

The overall survival probability of the restorations in the LD and ZrO_2_ groups after 1 year was evaluated. Direct intraoral clinical examination was carried out by two calibrated examiners independent of the investigation (Cohen’s Kappa 0.76). The double-blind evaluation was performed. The restorations were clinically observed under 20× magnification (Seiler, Mitron Instrument Revelation, St. Louis, MO, USA).

The quality of the restorations was evaluated according to modified USPHS criteria (United State Public Health Codes and Criteria) (Table 3) [15]. Immediate occlusal evaluation was carried out after bonding. The minor adjustments then considered necessary were performed. The tightness of the interproximal contact was verified using metal strips of 50 µm (Shimstock-Folie, Coltene, Altstätten, Switzerland) placed between the inlay and the adjacent tooth. At 2 weeks, 4 weeks, 6 months and 1 year, follow up evaluations were performed [15]. If any difference was found between both examiners, a third calibrated examiner (Cohen’s Kappa 0.76) established the final decision.

### 2.8. Statistical Analysis

Statistical analysis was performed using IBM SPSS statistics 24.0, SPSS (South Asia PVT LTD., www.spss.co.in, India, accessed on 27 December 2019). Comparison of the mean age by group was carried out following independent sample *t*-test. The categorical variable of gender was tabulated using a frequency procedure. The chi-square test was used to assess the association of groups, the association of anatomic deformity at follow-up visits with restorative materials in groups and the failure and the survival rate of restorations. A *p* value less than 0.05 was considered significant.

## 3. Results

The survival probability in the ZrO_2_ group amounted up to 93%, while in the LD group, this was 100%. The difference between groups was statically insignificant (Table 4). One restoration debonded completely in the ZrO_2_ group (class II MO, molar) just before the completion of one year of service.This restoration exhibited flaws (open/absent occlusal and proximal contacts, discontinuous with the existing anatomy, evidence of a positive step at margin, slightly pitted surface, and mild postoperative sensitivity) during all follow up-periods (2, 4 weeks and 6 months) (Table 5, Table 6, Table 7, Table 8, Table 9 and Table 10, marked with *)

In the ZrO_2_ group, the mean patient age amounted up to 36.27 ± 9.48 years, while in the LD group, this was 36.93 ± 8.65 years, and there was an insignificant difference between these values (*p* = 0.842). In both groups, the male to female ratio of 60% and 40% was found to be absolute matching (*p* = 1.0000).

Occlusal evaluation was carried out after bonding. Any necessary adjustments were performed, and the majority were minor. The ZrO_2_ group exhibited 80% normal occlusal and interproximal contact, while in LD group, this was 66.7% at all follow-up periods of 2 weeks, 4 weeks, 6 months and 1 year. However, 6.7% cases in ZrO_2_ showed open contact at all follow-up visits after 2 weeks, 4 weeks, 6 months and 1 year. The difference between groups was statically insignificant at all follow-up visits (Table 5).

There was no significant difference between the anatomical form in both groups for the anatomy of inlays (Table 6). The ZrO_2_ group remained continuous only in 73.3% cases, whereas 100% of the restorations in the LD group exhibited proper anatomic form.

In the ZrO_2_ group, 73.3% of cases exhibited closely adapted margins at the 2- and 4-week follow-ups. This percentage decreased to 66.7% after 6 months to 1 year. Whereas, in the LD group, 80% of restorations were closely adapted. However, the difference between groups was statically insignificant at all follow-up visits (Table 7). In the ZrO_2_ group, four restorations (26.7%) had a visible crevice; however, the sharp point of a probe (point diameter 0.5 mm, GDC Exs6XL, India) could not penetrate it. Moreover, one restoration had evidence of a step when the probe was drawn from the tooth for the restoration.

In the LD group, all restorations exhibited a smooth surface at all time intervals. In the ZrO_2_ group, this feature was observed for 93.3% of cases after 2 weeks, 4 weeks and 6 months. However, after 1 year, this value decreased to 80%. In contrast, in the LD group, only three restorations (20%) exhibited visible evidence of a crevice, but a sharp pointed probe was not able to penetrate even after one-year of follow-up. The difference between groups was statically insignificant at all follow-up visits (Table 8).

In the LD group, all restorations exhibited proper colour and translucency match, while in the ZrO_2_ group only 26.7% matched the colour of the tooth being restored. The difference between groups was statistically significant post-immediate placement of the restoration at all follow-up visits (Table 9).

Post-cementation of three restorations (20%) in group ZrO_2_ patients experienced mild, but bearable sensitivity at all follow-up time periods up to one year. However, the difference between groups was statically insignificant at all follow-up visits (Table 10).

## 4. Discussion

In this investigation, the null hypothesis was accepted. There was no difference between the clinical performance of the CAD-CAM zirconia dioxide and lithium disilicate inlays. It is worth emphasizing that this is the first clinical study on posterior indirect inlays comparing two different ceramic materials with a one-year follow-up.

It was evident that, due to the debonding of one restoration in the clinical scenario in group ZrO_2_, this group showed a slightly lower survival rate than did the LD restorations (93% and 100%, respectively) although this finding was not statistically significant. The present results are in agreement with other studies that evaluated partial coverage restorations and crowns [19]. The high survival probability of the restorations in the LD group could be a result of micromechanical and chemical bonds to the etched silica [20,21,22,23,24,25,26]. Consequently, micromechanical interlocking between the rough surface of the restoration and resin-based cement is created, which enhances the bond strength [27].

In addition, chemical bonds can be increased by silanization of the restoration bonding surface. Silane forms strong siloxane linkages between the restoration and resin interface [4]. The silane agent used in the present study was Monobond N (Monobond N, Ivoclar Vivadent Schaan, Liechtenstein, Germany), which is composed of three different functional monomers, namely silane methacrylate, phosphoric methacrylate and sulphide methacrylate [4,28]. However, in the ZrO_2_ group, one restoration (7%) debonded due to adhesive failure within two months. The remnants of the adhesive cement were located on the tooth surface. The adhesive procedure (Monobond N) of the CAD-CAM inlays did not result in a chemical bond to the ZrO_2_ restoration [29].

The traditional silanization is not effective in the case of restorations lacking a glass phase [30]. On the contrary, it was proven that the addition of MDP (methacryloyloxydcyl dihydrogen phosphate) to silane or to primer enhanced the bond strength of resin materials to zirconium oxide-based restorations [31,32,33,34]. MDP is a monomer derived from the reaction of methacrylic acid with phosphoric acid or carboxylic acid. It creates chemical (P = O, OH = Zr) or ionic bonds with ZrO_2_ [35].

Another possible reason for the debonding of two ZrO_2_ restorations could be due to the poor adhesion of this cement system (Multilink N, Ivoclar Vivadent Schaan, Liechtenstein) to dentine. The resin system either led to partial demineralization of the dentine substrate or to incomplete polymerization of the adhesive and cement, resulting in premature degradation of the interface [27,29,33,35,36,37,38,39,40,41]. The survival rates for all-ceramic restorations were found to be over 90% after 10 years of service [42].

In the present study, the ZrO_2_ (80%) and LD (66.7%) restorations exhibited and maintained normal occlusal and interproximal contacts at all follow-up periods up to one year. No statistical difference was observed. A similar outcome was seen in another study where there was no significant difference between LD and ZrO_2_ full-coverage crowns regarding the marginal, axial and occlusal fit [43]. Open proximal contact can contribute to, for instance, the formation of periodontal pockets, gingival inflammation, or proximal caries [44]. This can occur due to imperfections in impressions (traditional or digital), during fabrication (firing or sintering) of the ceramics or through wear at the interproximal surface [45].

The anatomical form in both the ZrO_2_ (73.3%) and LD (100%) groups remained continuous. The present study also evaluated the marginal adaptation of the restorations. Only 66.7% of restorations in the ZrO_2_ group and 80% in the LD group exhibited close marginal adaptation with no evidence of a catch or crevice up to the one-year follow-up. These findings are supported by several studies [45,46,47,48,49,50,51,52,53,54,55,56]. The most probable reason for visible crevices (26.7% after 1 year) in the ZrO_2_ group could be that ceramic veneering and layering on zirconia copings may result in an increased marginal gap compared with press techniques [56,57,58].

The marginal fit is one of the factors influencing possible restoration failure due to secondary caries and retention loss [59]. The marginal discrepancies can be observed due to the dissolution of luting cement, polymerization shrinkage of cement, occlusal load, type of finish line and margin placement (supra-gingival, sub-gingival or crestal gingival margin), salivary pH and brushing technique [60]. Moreover, the marginal gap can accumulate bacterial plaque and consequently result in carious lesions [57].

The clinical acceptable marginal discrepancy between prosthodontic restoration and the prepared tooth surface is approximately 50–120 μm [61,62,63,64]. However, minor marginal discrepancies in an indirect restoration may be compensated by the dual cure resin composite luting system [65]. The present study used conventional impressions in the LD group and digital ones in the ZrO_2_ group according to the recommendations of other studies [42,66,67]. The internal fit of restorations was proven to be comparable for both impression techniques [67].

In the LD group, all cases presented a smooth surface up to one year. This finding is in consensus with similar clinical studies and laboratory studies that evaluated the surface smoothness of all ceramic restorations [2,12,15,16,42,48,66,68,69,70,71,72]. In the ZrO_2_ group, 13.3% inlays exhibited surface fracture/chipping of the veneering ceramic after one year. These results are supported by similar studies that evaluated the clinical chipping of porcelain from zirconium dioxide substructures [19,73]. The crack formation and propagation occurs when the tensile strength within the ceramic exceeds the tensile strength of the veneering ceramic [68].

The tensile strength of the ceramic is the sum of the external and residual stresses. Without any load applied, residual stress persists, which can cause immediate or delayed ceramic cracks. On the contrary, external stress is formed within the structure by externally applied loads that occur during function and mastication [42,69]. Moreover, LD ceramic has an extended microcrystal structure (3–6 µm), which provides a strong bond with tooth structure after cementation [3,4,5,6,49]. This structure perfectly distributes forces due to the increased surface area of the crack and the interlocking microstructure of the ceramic. The crack propagation is described as an intragranular process and is characterised by a meandering line. Thus, the spread of a crack through this material is stopped by lithium disilicate crystals, providing a substantial increase in the bending strength and fracture toughness [70].

In the present study, all the LD group cases matched the colour and translucency, whereas in the ZrO_2_ group, the matches amounted to up to 26.3% of cases. The clinical evaluation of the surface and colour of the LD crowns (Empress 2) after 14 years was found to be in the range of excellence [64,71,72,74,75,76]. The perfect aesthetics outcome of LD restorations were in accordance with several other studies [8,12,15,16,19,61,63,70,71]. The reasons for the high aesthetics of LD restorations are polyvalent ions in the glass that provide the desired colour, the even distribution of glass ceramics with leucite and lithium-disilicate-reinforced crystals in the single-phase equipment and the elimination of pigment defects in the microstructure [67].

Moreover, the similar light refraction index between glass ceramics with leucite and lithium-disilicate-reinforced crystals leads to high translucency [49]. However, in some cases, the complex optical characteristics of tooth colour makes it difficult to achieve a close shade match of an artificial restoration to the natural tooth structure [25]. On the contrary, ZrO_2_ is white in colour and opaque. In the ZrO_2_ group, all restorations were performed with single blocks of the same colour and opacity, which may have hampered the ability to mimic a natural appearance.

Therefore, this procedure does not always provide an optimal aesthetic integration, and consequently a veneering material should be applied [72]. In the present study, a glaze was applied to increase the gloss of the restorations, and tints were used to mimic the pits and fissures. The glaze resulted in a darker appearance of some restorations at the baseline recall, but the glaze was mostly lost after 1 year. A decrease in the translucency of some restorations was observed.

Additional reasons for a poor colour match could be the repeated firing of all ceramic zirconia cores and the thickness of the dentine porcelain [73,76]. Certain metal oxides are not colour stable after they are subjected to firing temperatures due to pigment breakdown of surface colorants [25,28,30]. Additionally, visual shade selection could contribute to the colour mismatch. However, several studies found no difference between visual and instrumental shade selection techniques [42,68,69,70,72].

In this study, all patients in the LD group and 80% in the ZrO_2_ group did not report post-operative sensitivity at all follow-up periods. However, there was no significant difference in sensitivity between the two groups in the follow-up period. Postoperative sensitivity has been attributed to several factors, including trauma due to dentin preparation, dentin etching, bacterial penetration of the pulp, occlusal discrepancies, the extent of cavity preparation, type of bonding, luting procedure and polymerization shrinkage [76,77]. A relatively low post-operative sensitivity rate was observed. A possible reason could be the mild-etching potential of the self-etch adhesive luting cement (Multilink N), which did not cause over-etching and created a uniform hybrid layer. These findings are in agreement with several studies showing a low or lack of post-operative sensitivity for restorations luted using a self-etch mode [19,72,73,74,75,78,79].

There are several in vitro and clinical studies comparing fixed prosthesis, including ceramic restorations, using different parameters [27,28,30,34,36,37,38,39,40,41,45,55,61,80]. Several clinical studies used USPHS criteria for tooth-coloured restorations in posterior teeth [15,66,78]. Therefore, this method was used to assess zirconia dioxide and lithium disilicate inlay restorations in the present study.

A low number of restorations was investigated in this study, and thus evaluations on larger study groups are needed. Moreover, only two ceramic materials and one adhesive agent and cement were used. Similar studies embracing more materials should be conducted in the future. There is a need to prolong the follow-up period to investigate both techniques in long-term studies. Additionally, the investigation was performed at one university, and thus more multicentre studies should be carried out; private dental offices should be also included to provide a wider perspective.

## 5. Conclusions

Within the imitations of the present study, the lithium disilicate- and zirconia dioxide-based inlays exhibited comparable clinical performance. However, the colour and translucency match was superior for the lithium disilicate restorations.

## Figures and Tables

**Table 1:** Inclusion and exclusion criteria of patients.

Inclusion Criteria	Exclusion Criteria
class II cavities in permanent teeth	severe systematic diseases and allergies
isthmus size of the treated cavities at least half of the	severe salivary gland dysfunction
intercuspal distance	severe periodontal problems
no clinical signs and symptoms of pulp and periapical pathology	poor plaque control
at least one neighbouring tooth	parafunctional habits like bruxism or clenching
in occlusion to antagonistic teeth	restricted mouth opening
good oral hygiene	history of orthodontic treatment
over 18 years old	preparations extending below the gingiva margin and close to the pulp
willing to participate in the study	initial defects, i.e., discoloured pits and fissures and caries restricted to enamel only

**Table 2:** The Class II mesio-occlusal (MO) and occluso-distal (DO) cavity distribution within study groups.

Tooth Type	Type of Class II Cavity	LD	ZrO_2_
Premolars	MO	4	5
	OD	3	2
Molars	MO	4	4
	OD	4	4
-Total no of teeth	30	15	15

**Table 3:** The post-operative review assessment codes and criteria-USPHS criteria.

Assessment Criteria	Parameters
(1) Occlusal and interproximal contact	(A) Normal
	(B) Heavy
	(C) Light (D) Open
(2) Anatomic form	(A) Continuous with existing anatomy (B) Discontinuous with existing anatomy, but not sufficient to expose dentine/base exposed (C) Dentine/base exposed
(3) Marginal adaptation	(A) Closely adapted no evidence of a catch or crevice at any point (B) Visible evidence of a crevice. Fine probe will not penetrate (C) Visible evidence of a crevice. Fine probe will penetrate (D) Evidence of a positive step when probe drawn from tooth to restoration
(4) Surface roughness	(A) Smooth
	(B) Slightly pitted
(5) Colour Match	(A) Matches colour and translucency of adjacent tooth structure. (B) Mismatch in colour and translucency is within the acceptable range
(6) Sensitivity	(A) None (B) Mild but bearable (C) Uncomfortable (D) Very painful data
(7) Overall survival probability of restorations after one year	(A) In percentage

**Table 4:** The survival probability in the study groups.

Study Group	Survival Probability	
	No.	%
LD	15	100.0
ZrO_2_	14	93.0
Total	29	96.0
Chi- square and *p* value	χ^2^ = 5.9032; *p* = 0.522	

**Table 5:** Occlusal and proximal contact in study groups.

Follow-Up Periods	Occlusal and Proximal Contact	Group (χ^2^ = 4.612, *p* = 0.242)					
		LD		ZrO_2_		Total	
		No.	%	No.	%	No.	%
2 weeks	Normal	10	66.7	12	80.0	22	73.3
4 weeks	Heavy	3	20.0	0	0	3	10.0
6 months	Light	2	13.3	2	13.3	4	13.3
1 year	Open/Absent	0	0	1 *	6.7	1	3.3

* One inlay was lost just before completion of the 1-year evaluation.

**Table 6:** The anatomic form in the study groups.

Follow-Up Periods	Anatomic Form	Group (χ^2^ = 4.615, *p* = 0.032)					
		LD		ZrO_2_		Total	
		No.	%	No	%	No.	%
2 weeks	Continuous with the existing anatomy	15	100.0	11	73.3	26	86.7
4 weeks 6 months 1 year	Discontinuous with the existing anatomy but not sufficient enough to expose dentin/base	0	0	4 *	26.7	4	13.3

* One inlay was lost just before completion of the 1-year evaluation.

**Table 7:** The marginal adaptation in study groups.

Follow-Up Periods	Marginal Adaptation	Group (χ^2^ = 1.043; *p* = 0.593)					
		LD		ZrO_2_		Total	
		No.	%	No	%	No.	%
2 weeks 4 weeks	Closely adapted. No evidence of a catch or crevice at any point	12	80.0	11.0	73.3	26.0	86.7
	Visible evidence of a crevice. Fine probe will not penetrate	3	20.0	3.0	20.0	6.0	20.0
	Visible evidence of a crevice. Fine probe will penetrate	0	0	0	0	0	0
	Evidence of a positive step when probe drawn from tooth to restoration	0	0	1.0	6.7	1.0	3.3
6 months 1 year	Closely adapted. No evidence of a catch or crevice at any point	12	80.0	10.0	66.7	22.0	73.3
	Visible evidence of a crevice. Fine probe will not penetrate	3	20.0	4.0	26.7	7.0	23.3
	Visible evidence of a crevice. Fine probe will penetrate	0	0	0	0	0	0
	Evidence of a positive step when probe drawn from tooth to restoration	0	0	1.0 *	6.7	1	3.3

* One inlay was lost just before completion of the 1-year evaluation.

**Table 8:** The surface roughness in the study groups.

Follow-Up Periods	Surface Roughness	Group (χ^2^ =1.034; *p* = 0.309)					
		LD		ZrO_2_		Total	
		No.	%	No	%	No.	%
2 weeks 4 weeks 6 months	Smooth	15	100	14	93.3	29	96.7
	Slightly pitted	0	0	1	6.7	1	3.3
	Deeply pitted	0	0	0	0	0	0
	Surface fractured	0	0	0	0	0	0
1 year	Smooth	15	100	12	80	27	90
	Slightly pitted	0	0	1 *	6.7	1	3.3
	Deeply pitted	0	0	0	0	0	0
	Surface fractured	0	0	2	13.3	2	6.7

* One inlay was lost just before completion of the 1-year evaluation.

**Table 9:** The colour match in the study groups.

Immediate Colour Match	Groups (χ^2^ = 17.368, *p* = 0.000)					
	LD		ZrO_2_		Total	
	No.	%	No.	%	No.	%
Matches colour and translucency of adjacent tooth structure	15	100	4	26.7	19	63.3
Mismatch in colour and translucency	0	0	11	73.3	11	36.7

**Table 10:** The occurrence of sensitivity in the study.

Follow-Up	Sensitivity	Groups (χ^2^ = 3.33, *p* = 0.068)					
		LD		ZrO_2_		Total	
		No.	%	No.	%	No.	%
2 weeks	None	15	100	12	80	27	90
4 weeks	Mild but bearable	0	0	3 *	20	3	10
6 months	Uncomfortable	0	0	0	0	0	0
1 year	Very painful	0	0	0	0	0	0

* One inlay was lost just before completion of the 1-year evaluation.

## Data Availability

The data presented in this study are available on request from the corresponding author.
